# Estimating the impact of airport wildlife hazards management on realized wildlife strike risk

**DOI:** 10.1038/s41598-024-79946-3

**Published:** 2024-11-22

**Authors:** Levi Altringer, Michael J. Begier, Jenny E. Washburn, Stephanie A. Shwiff

**Affiliations:** 1 US Department of Agriculture, Animal and Plant Health Inspection Service, Wildlife Services, National Wildlife Research Center, Fort Collins, 80521 CO USA; 2 US Department of Agriculture, Animal and Plant Health Inspection Service, Wildlife Services, Airport Wildlife Hazards Program, Washington, 20250 DC USA; 3 US Department of Agriculture, Animal and Plant Health Inspection Service, Wildlife Services, Airport Wildlife Hazards Program, Sandusky, 44870 OH USA

**Keywords:** Aviation, Airports, Wildlife-aircraft collisions, Wildlife hazards management, Causal inference, Environmental economics, Natural hazards

## Abstract

Collisions between wildlife and aircraft, commonly referred to as wildlife strikes or bird strikes, are rare events that pose considerable safety and economic risks to the aviation industry. Given the potentially dramatic consequences of such events, airports scheduled for passenger service are required to conduct wildlife hazard assessments and implement wildlife hazard management plans for the purpose of mitigating wildlife strike risk. The evaluation of such management, however, is complicated by imperfect reporting that mediates the relationship between realized wildlife strike risk and wildlife strike metrics. In this paper, we shed light on such phenomena by investigating the staggered adoption of a federal wildlife hazards management program at joint-use airports across the contiguous United States. This research design allowed us to exploit variation in both management presence across airports, over time as well as variation in the quality of wildlife strike reporting within airports. As hypothesized, we found that wildlife hazards management intervention has a significant impact on the quality of reporting, as evidenced by a substantial increase in the number of civil strikes reported over the management period. Where pre-existing reporting mechanisms were more robust, however, we found that wildlife hazards management had a significant impact on realized wildlife strike risk as evidenced by a decrease in strike-induced economic damages among military aircraft. Overall, we found that the estimated economic benefits of the studied airport wildlife hazards management program were 7 times greater than the costs over the management period. Our results have important implications for the measurement of wildlife strike risk and the management of wildlife hazards at airports, as well as important insights pertaining to the use of observational data for causal inference, particularly in the context of risk management.

## Introduction

Wildlife-aircraft collisions, commonly referred to as wildlife strikes or bird strikes, are relatively rare events that pose considerable safety and economic risks within the aviation industry. Damaging wildlife strike events generate substantial repair costs as well as downtime for aircraft and commercial aircraft passengers^1–5^. In the most severe and rare instances, wildlife strikes can cause injury and even loss of life^6^. Given that roughly 75 percent of such incidents occur at $\le$ 500 feet AGL^7^, airports certified for passenger service are often required to alleviate wildlife hazards whenever detected^8–11^. This includes conducting wildlife hazards assessments, and, if necessitated by the wildlife hazards assessment, developing and maintaining a wildlife hazards management plan to mitigate wildlife strike risk.

**Fig. 1:**
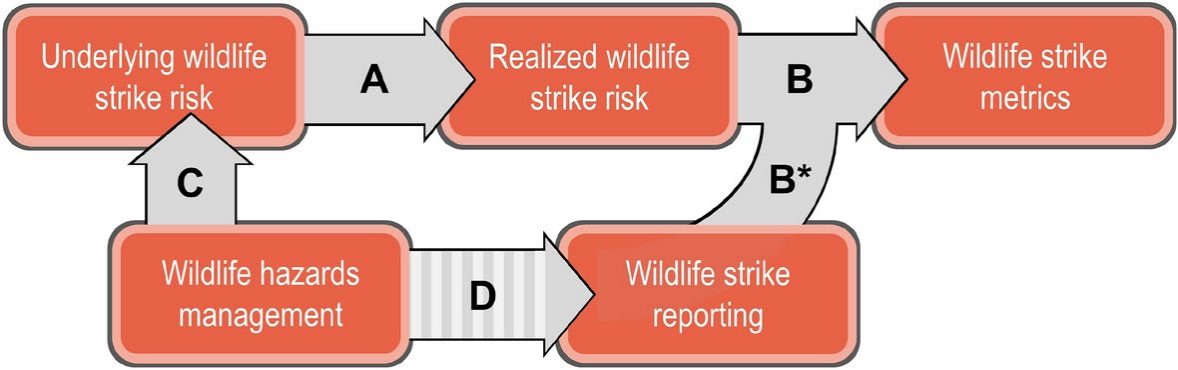
Conceptualizing the relationship wildlife strike risk, wildlife strike metrics, and the hypothesized impact of airport wildlife hazards management.

Wildlife strike risk is a term used to summarize the threat wildlife pose to aircraft along two dimensions; (1) the likelihood of a wildlife strike incident—i.e., frequency—and, given its occurrence, (2) the likelihood that such an incident results in a negative outcome—i.e., severity^12–14^. Wildlife strike risk is influenced by a multitude of ecological and biological factors that affect the presence, abundance, and behavior of wildlife in proximity to airports and aircraft^15–22^. The objective of wildlife hazards management at airports, then, is to manage such factors so as to the limit the frequency and severity of wildlife strike incidents^17,23,24^. While the objective of airport wildlife hazards management is clear, its evaluation is not straightforward. This is primarily due to the fact that wildlife strike risk exists as a latent variable^25^. That is, there exists no directly observable measure that is wildlife strike risk and, instead, the phenomenon can only be inferred through other variables that can be observed and measured. We adopt the term *underlying wildlife strike risk* to emphasize the latency of the phenomenon (Fig. 1).

Generally, there are two approaches to measuring underlying wildlife strike risk at an airport. First, one can proxy for the phenomenon by synthesizing observed ecological and biological variables that are hypothesized to constitute wildlife strike risk^20^. Second, underlying wildlife strike risk can be inferred *ex post* from wildlife strike incidents, or what we term *realized wildlife strike risk* (Fig. 1A)^12,14,26^. When attempting to evaluate the impact of wildlife hazards management on wildlife strike risk at airports, the first approach is limited by the availability of data. Specifically, the ecological and biological measures necessary for this approach are not historically available and, further, do not exist at the spatial scale required to evaluate changes in wildlife strike risk at a given airport. Realized wildlife strike risk, as measured through wildlife strike records that detail the frequency and severity of wildlife strike incidents at the airport-level, supplies the only practical means to evaluate airport wildlife hazards management^27^.

Using measures of realized wildlife strike risk—i.e., *wildlife strike metrics* (Fig. 1B)—to evaluate management programs at airports, however, does not come without limitations. This is because wildlife strike metrics rely on records of wildlife strike incidents that may be incomplete, both in quantity and quality. The robustness of *wildlife strike reporting* determines the extent to which wildlife strike metrics provide an accurate measure of realized wildlife strike risk (Fig. 1B*). Consider the often-used wildlife strike rate, which is the number of wildlife strike incidents per aircraft movement at an airport over a given time period—e.g., 1 strike per 5,000 aircraft movements. All else equal, an airport with a low reporting rate will have lower wildlife strike rate when compared to an airport with a higher reporting rate. Next, consider the *damaging* wildlife strike rate, which is the number of *damaging* wildlife strike incidents per aircraft movement in a given time period. The damaging wildlife strike rate is determined by both the number of wildlife strike reports as well as the number of reports that provide information on whether the aircraft experienced strike-induced damage. As the reporting of damage improves, all else equal, the damaging wildlife strike rate grows. In both examples, improvements in the quantity and quality of reporting reflect increases in the frequency and severity of wildlife strike incidents—i.e., wildlife strike risk. However, it is changes in reporting that are causing the observed changes in the wildlife strike metrics, not changes in risk. Consequently, wildlife strike reporting plays a pivotal role in influencing the reliability of wildlife strike metrics as measures of underlying and realized wildlife strike risk.

The primary objective of wildlife hazards management at airports is to mitigate wildlife strike risk, thereby reducing the frequency and severity of wildlife strike incidents^17^. The effectiveness of management intervention depends on the quality and intensity of the management practices (Fig. 1C). In so far as management-induced changes in wildlife strike metrics reflect changes in underlying and realized wildlife strike risk, the evaluation of management programs at airports is relatively straightforward. However, such evaluations become dubious if wildlife hazards management intervention not only impacts wildlife strike risk but also affects wildlife strike reporting (Fig. 1D). For instance, the presence of wildlife biologists at airports, engaging with operations personnel and conducting outreach and awareness campaigns, is likely to influence the quantity and quality of reported wildlife strike incidents. In this scenario, it becomes challenging to disentangle potential changes in realized wildlife strike risk from coincident alterations in wildlife strike reporting, both of which are attributable to management intervention. The relationship between airport wildlife hazards management and wildlife strike reporting is presented as uncertain—a dashed arrow (Fig. 1D). This is because we hypothesize that the ability of management programs to influence wildlife strike reporting at a given airport is determined by the pre-existing integrity of wildlife strike reporting. For instance, if the reporting of wildlife strike incidents at an airport is far from perfect, then the potential impact of a management program on the quantity and quality of reporting could be significant. Conversely, if wildlife strike reporting at an airport is excellent, then the potential impact of a management program on reporting will be minimal. Based on this reasoning, we postulate that the ability to ascertain the impact of management on realized wildlife strike risk increases with the integrity of pre-management reporting systems.

**Fig. 2:**
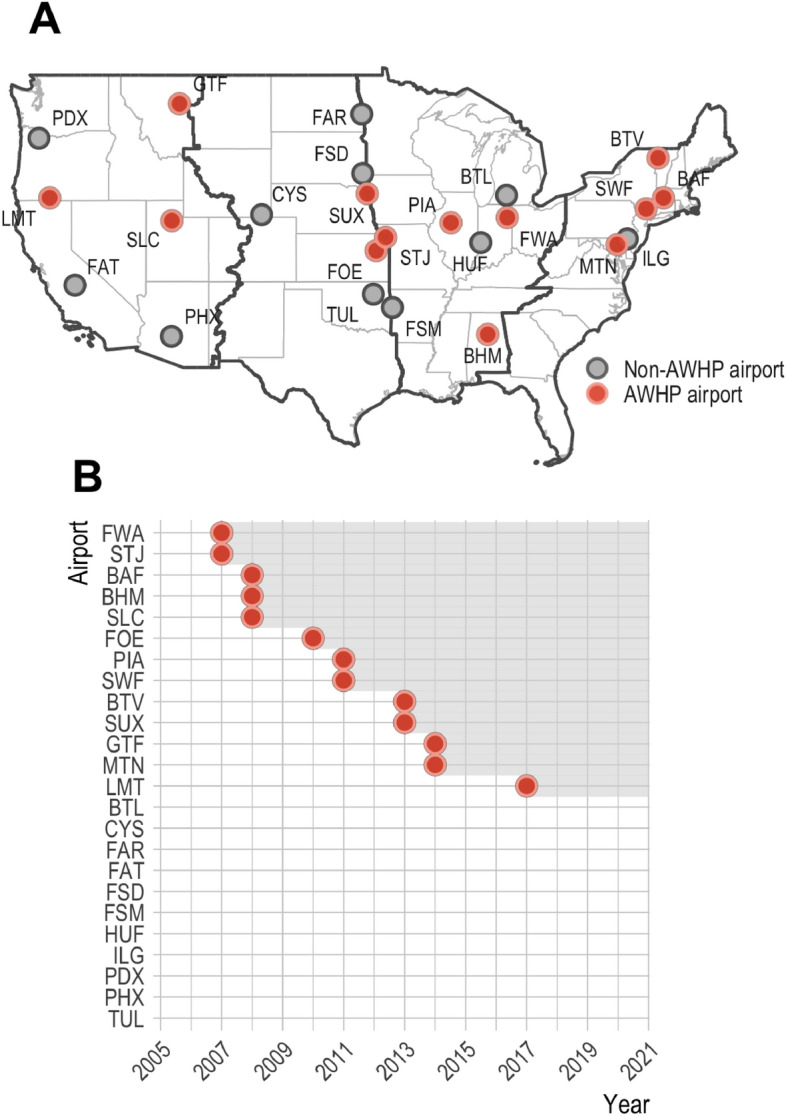
The location (**A**) and timing (**B**) of AWHP management intervention among sample airports.

Given all of the aforementioned complications, many have opted for evaluating wildlife hazards management through case studies, wherein species-specific management strategies are assessed based on species-specific outcomes over short time horizons^28–33^. While these studies crucially contribute to the formulation and execution of effective wildlife hazards management plans, they often do not provide the comprehensive program evaluation desired by airport managers and policymakers^27^. The goal of this study was to fill this gap through a strategic research design that disentangles the hypothesized impacts of airport wildlife hazards management on both wildlife strike reporting and risk. Specifically, we investigated the adoption of Airport Wildlife Hazards Program (AWHP) management at joint-use airports across the contiguous United States (Fig. 2). The AWHP is a national program contained within the United States Department of Agriculture’s Wildlife Services (USDA-APHIS-WS) that collaborates with the Federal Aviation Administration (FAA), Department of Defense, and the National Association of State Aviation Officials through memorandums of understanding to provide federal leadership and expertise in managing airport wildlife hazards. This includes educating airport personnel, training wildlife biologists, and directly managing wildlife hazards at airports across the United States. The wildlife hazards management conducted by the AWHP is performed by certified wildlife biologists and includes: aircraft flight schedule modification (if possible), habitat modification and exclusion, repellent and harassment techniques, and wildlife removal. As of 2022, the AWHP provided 343 staff-years of assistance at 813 airports, with 158 of these airports receiving full-time assistance—i.e., > 1 staff year.

Through a comparison of non-AWHP and AWHP-managed airports in both the pre-management and management periods, we investigated the impact of AWHP management on realized wildlife strike risk and wildlife strike reporting through relative changes in wildlife strike metrics. We focused on three wildlife strike metrics in this study: the overall wildlife strike rate, the disruptive wildlife strike rate, and wildlife strike costs. These metrics, each in their own way, proxy for changes in the frequency and severity of wildlife strike incidents^14,27^. Our study specifically targeted joint-use airports, which functioned as civil airports and Air National Guard bases over the sample period—accommodating general aviation, air carrier, air taxi, and military aircraft movements. This dual usage allowed us to examine wildlife strike metrics as joint, civil-, or military-specific measures. The disaggregation of wildlife strike metrics into their civil and military components allowed us to analyze the effects of AWHP management under heterogeneous reporting systems, since civil and military wildlife strike incidents are recorded in separate, independent databases. Civil incidents are reported to the National Wildlife Strike Database through a decentralized, largely voluntary system^6,34,35^. In contrast, wildlife strikes involving Air National Guard aircraft are documented in the Air Force Safety Automated System^36^, a centralized and accountable system. Below, we provide evidence that the quantity and quality of wildlife strike reporting within the Air National Guard is relatively robust compared to the reporting of civil wildlife strike incidents. Henceforth, all Air National Guard aircraft movements and related wildlife strike incidents will be collectively categorized and referred to as *military* aircraft movements and wildlife strike incidents.

Before moving to our methods and results, it is important to note here that our study does not compare unmanaged and managed airports. While such a research design is preferable, it was infeasible due to FAA regulations that require airports certified for passenger service to conduct wildlife hazards assessments and alleviate wildlife hazards whenever detected^9^. In other words, non-AWHP airports are likely to have some level of wildlife hazards assessment and management. The absence of management documentation among non-AWHP airports, however, disallowed any measurement of the timing or intensity of potential non-AWHP management activities. Instead, our study compared those airports that adopted AWHP management to non-AWHP airports. Our *a priori* expectation was to find significant management effects among AWHP-managed airports. The underlying assumption required by this expectation is that AWHP management, through its institutional resources and expertise, provided more robust wildlife hazards management upon intervention. Beyond anecdotal evidence, there is no way to empirically test this assumption. However, in our analysis, we differentiated between low- and high-AWHP management airports to test the extent to which estimated management effects ordered on management staffing. Investigating the heterogeneous impact of management intervention among AWHP-managed airports provides an estimate of airport wildlife hazards management more generally. In any case, the results of this study were carefully interpreted as the estimated impact of AWHP management, specifically, with implications airport wildlife hazards management, generally.

## Methods

### Data

The timing, location, and staffing of AWHP management was derived from program administrative files as well as management task records collected from the USDA-APHIS-WS Management Information System (MIS)^37^. The wildlife strike metrics employed in our analysis were constructed from civil and military wildlife strike records collected from the National Wildlife Strike Database (NWSD)^35^ and the Air Force Safety Automated System (AFSAS)^36^, respectively. The civil and military databases provide comprehensive details on each recorded wildlife strike incident, including the date and location of the strike, the species involved, the impact on the flight, the extent of damage, and the cost of repairs, among many other relevant pieces of information. In addition to AWHP management and wildlife strike records, civil and military aircraft movement—i.e., takeoffs and landings—data were collected from the FAA’s Air Traffic Activity Data System (ATADS)^38^.

The sample of airports and years employed in our study was determined by the conditions of our research design. First, to compare non-AWHP and AWHP-managed airports in both the pre-management and management periods, we needed to observe the precise timing of AWHP management intervention and needed sufficient data coverage prior to and following management intervention. Second, to investigate the impact of AWHP management intervention on both civil- and military-specific wildlife strike metrics, we restricted our sample to include only joint-use airports. Lastly, our sample was restricted by the availability of civil and military aircraft movement data, which is an essential piece of information when assessing changes in the frequency of wildlife strike incidents across airports, over time. Constrained by the previously mentioned criteria, we utilized management records from the AWHP and Air National Guard to identify joint-use airports that had and had not received AWHP management over the 2005 to 2021 period. This resulted in a panel of 24 airports—13 AWHP-managed airports and 11 non-AWHP airports—that provide representation across the contiguous United States and North American migratory flyways (Fig. 2). In our sample, the average AWHP-managed airport employed approximately 0.97 (SD = 0.418) AWHP management staff years annually over the management period. Three sample airports stood out as distinct from the others—Portland International Airport (PDX), Phoenix International Airport (PHX), and Salt Lake City International Airport (SLC). These airports are operationally large relative to the others in the sample and, therefore, could have a disproportionate influence on sample means and estimated management effects. We conducted sensitivity analyses in the Supplementary Information to ensure that our model estimated management effects were robust to the inclusion or exclusion of these three airports.

### Measurement

Between 2005 and 2021, our sample of 24 airports accounted for 9,389 civil wildlife strike records in the NWSD and 2,511 military records in the AFSAS. Not all wildlife strike records provided the exact species involved, but included the species family or order—e.g., Ring-billed gull (*Larus delawarensis*); Gull, tern, or kittiwake (*Laridae*); or Shorebird (*Charadriiformes*)—while other records provided no specific information. Across civil and military wildlife strike records, a total of 388 unique species, families, or orders were recorded. The most commonly struck species in the sample were horned lark (*Eremophila alpestris*) ($n = 846$), American kestrel (*Falco sparverius*) ($n = 646$), and mourning dove (*Zenaida macroura*) ($n = 630$). Mammals, excluding bat species, accounted for only 0.9 percent ($n = 105$) of sample wildlife strike records—2 of these records were collisions with white-tailed deer (*Odocoileus virginianus*). Among civil wildlife strike records, 28 percent ($n = 2,636$) provided no species information. Alternatively, only 7 percent ($n = 185$) of military wildlife strike records were missing species information.

The mean number of reported wildlife strike incidents for the average airport in our sample was 29.17 (SD = 39.35) per year, with an average 23.01 (SD = 38.65) and 6.15 (SD = 7.75) attributed to civil and military records, respectively. The difference between civil and military wildlife strike incidents is largely attributable to differences in aircraft movements across the two groups (Supplementary Information Table S1). The overall wildlife strike rate, our first measure of interest, is calculated as the total number of wildlife strikes reported at an airport in a given year, divided by the total number of aircraft movements at that airport in the same year. Wildlife strike rates are often multiplied by scaling factor that provides a more straight-forward interpretation—our study uses a factor of 5,000. The overall wildlife strike rate serves as a crude measure of wildlife strike risk, offering insights primarily focused on the frequency of wildlife strike incidents. Among the airports in our sample, the joint overall wildlife strike rate averaged 1.87 (SD = 2.01) per 5,000 aircraft movements annually. When disaggregating wildlife strikes and aircraft movements into their civil and military components, average overall wildlife strike rates were 1.41 (SD = 1.80) and 5.30 (SD = 7.58), respectively.

Disruptive wildlife strikes are those strike records that indicated repair costs, non-repair (other) costs, damage, a negative effect on the flight, aircraft downtime, or any combination of these. Among the wildlife strike incidents contained within our sample, 904 civil and 725 military wildlife strike records were classified as disruptive. The average number of disruptive wildlife strike incidents per year for a typical airport in our sample was 3.99 (SD = 4.77), with civil and military records contributing an average 2.22 (SD = 3.82) and 1.78 (SD = 3.03) disruptive strike incidents per year, respectively. The disruptive wildlife strike rate is calculated similarly to the overall strike rate, but provides a more comprehensive measure of wildlife strike risk that reflects changes in both the frequency and severity of wildlife strike incidents^14,27^. The mean disruptive wildlife strike rate for the average airport in our sample was 0.32 (SD = 0.46) per 5,000 aircraft movements, with civil-specific and military-specific rates of 0.13 (SD = 0.16) and 1.42 (SD = 2.64) per 5,000 aircraft movements, respectively.

Costs associated with wildlife strike incidents were taken directly from the costs disclosed within civil and military strike records. On the civil side, costs are reported as “repair” and “other” costs in the NWSD, with total reported wildlife strike costs being calculated as the sum of these two measures. Alternatively, in the AFSAS, costs are reported as the total mishap cost either including or excluding injury costs. Since injury costs are not included in civil wildlife strike records, and to maintain some consistency across civil and military strike records, we used total mishap costs that do not include injury costs among military strike records. Over the sample period, the total cost of wildlife strikes amounted to $158.9 million (2023 $). Hereafter, all cost figures and estimates are measured in millions of 2023 dollars unless otherwise indicated. The distribution of annual wildlife strike costs was positively skewed, characterized by numerous years of no costs and a few years with exceptionally high costs. Joint strike-induced costs averaged $0.39 (SD = $1.89) annually for the average airport in our sample. Broken down by sector, average annual costs were $0.15 (SD = $0.79) among civil records and $0.24 (SD = $1.66) among military records. For those airport-year observations with non-zero wildlife strike costs, average annual strike-induced costs were $0.75 (SD = $2.56) jointly, $0.53 (SD = $1.42) among civil wildlife strike records, and $0.73 (SD = $2.85) among military strike records.

Before moving to our estimation strategy, it is important to note the significant difference in civil- and military-specific wildlife strike metrics among sample airports. Reporting of species information was much greater among military strike records—93 percent among military records compared to 72 percent among civil records. The military-specific overall wildlife strike rate was roughly 4x larger than the civil-specific overall wildlife strike rate while the military-specific disruptive wildlife strike rate was over 10x larger than that among civil aircraft. Reported military wildlife strike costs were roughly 50 percent greater than civil-side costs despite the fact that there were 3x the number of civil strike records. Differences in aircraft and flight characteristics across civil and military aircraft notwithstanding^15,18^, the disparities observed above suggest that, within our sample of airports, the quantity and quality military wildlife strike reporting was relatively robust compared to civil wildlife strike reporting.

### Estimation strategy

Simple comparisons of outcomes across or within airports are inadequate to estimate the impact of AWHP management on realized wildlife strike risk. Cross-sectional comparisons are biased by the presence of airport-specific factors that are likely to be correlated with selection into AWHP management. Additionally, analysis of time series data for individual airports are likely biased by trends in wildlife strike risk and reporting that are common across all airports, regardless of management. The use of panel data techniques, however, which combine the cross-sectional and time series aspects of our data, allow for the simultaneous control of (1) time-invariant, airport-specific characteristics that are likely correlated with AWHP adoption and (2) trends in wildlife strike risk and reporting over time that are common across airports^39^.

Specifically, to estimate the impact of AWHP management intervention on realized wildlife strike risk, we employed a staggered difference-in-differences research design^39^. This strategic research design uses panel data techniques to compare the before-and-after change in an outcome among AWHP-managed airports with the contemporaneous change in outcomes among non-AWHP managed airports. It is the difference between these two differences, which is net of location-specific and general time influences, that provides the estimated impact of AWHP management. Difference-in-differences research designs are a common tool for causal inference in modern econometrics and they have been used to estimate policy and program effects across a variety of settings. Recent applications of such methods in the context of wildlife management include the impact of wolf recolonization on deer-vehicle collisions in Wisconsin^40^, the impact of bison reintroduction on local economies in the U.S.^41^, the impact of private versus government management on wildlife and tourism outcomes in Africa^42^, and the impact of badger control policy on bovine tuberculosis in England^43^.

The staggered difference-in-differences research design is estimated using a two-way fixed effects (TWFE) regression estimator that extends the well-known difference-in-differences approach to accommodate multiple treated units experiencing staggered intervention^44^, as is the case in our analysis (Fig. 2). Although the TWFE regression estimator has been widely employed to assess policy and program effects, recent methodological studies have highlighted its potential to produce misleading results^45,46^. Fortunately, several alternative estimators have been developed that avoid these pitfalls^47–51^. We employ one of these recently-developed robust estimators, but begin with a presentation of the traditional TWFE regression estimator because of its straightforward interpretation, which provides essential intuition for our estimation strategy.

The standard TWFE regression model is presented as follows

1 $$\begin{aligned} y_{it} = \beta D_{it} + \theta X_{it}' + \alpha _{i} + \delta _{t} + u_{it} \end{aligned}$$

where $y_{it}$ is the wildlife strike metric of interest measured at airport *i* in year *t*, $X_{it}$ is a vector of time-variant covariates, $\alpha _{i}$ and $\delta _{t}$ are airport and year fixed effects, respectively, and $u_{it}$ is the idiosyncratic disturbance term. The inclusion airport effects ($\alpha _{i}$), which are indicator variables for each airport, provides a nonparametric method to control for all time-invariant factors that could affect the mean differences in wildlife strike metrics across airports, without the need to specify each factor that could influence underlying wildlife strike risk and reporting at a particular airport. Similarly, the inclusion of year effects ($\delta _{t}$), which are indicator variables for each year, controls for time-variant factors that might affect trends in underlying wildlife strike risk and reporting that are common to all airports.

The variable of interest in Eq. 1 is $\text {D}{it}$, a dummy variable indicating the presence of AWHP management at airport *i* in year *t*. Since the initial year of AWHP management begins at different points within the calendar year for each airport—e.g., January versus October—$\text {D}_{it}$ is set equal to 1 starting from the first *full* year of management and thereafter, and 0 otherwise. For airports that did not receive AWHP management intervention, $\text {D}_{it}$ remains 0 for all periods. The coefficient associated with the management indicator, $\beta$, provides the estimate of interest. This estimated $\beta$ is typically referred to as the average treatment effect on the treated and represents, in the context of our research, the effect of AWHP management for the average AWHP-managed airport in the sample, averaged across all management years.

For each wildlife strike metric used in our analysis—the overall wildlife strike rate, disruptive wildlife strike rate, and wildlife strike costs—an estimated management effect that is negative—i.e., $\hat{\beta } < 0$—reflects a reduction in realized wildlife strike risk. For example, if AWHP management is estimated to have a negative impact on the overall wildlife strike rate, this suggests a decrease in the frequency of wildlife strike incidents and, therefore, and decrease in risk, all else equal. Similarly for the disruptive wildlife strike rate and strike-induced economic costs. We hypothesized that the impact of AWHP management on wildlife strike metrics would be twofold. First, we hypothesized that AWHP management intervention would reduce wildlife strike risk and, therefore, have a negative impact on wildlife strike metrics—i.e., decrease. Oppositely, however, we hypothesized that AWHP management intervention could improve the reporting of wildlife strike incidents and have a positive impact on wildlife strike metrics—i.e., increase.

The estimated net effect of AWHP management intervention on wildlife strike metrics, then, depends on the relative impacts of AWHP management on wildlife strike risk and reporting. If the risk-reducing effects of AWHP management are relatively large compared to management-induced improvements in reporting, then we would expect to observe estimated management effects that are negative—i.e., $\hat{\beta } < 0$. Alternatively, if management-induced reporting improvements are relatively large compared to the risk-reducing effects of AWHP management, we would expect to observe estimated management effects that are positive—i.e., $\hat{\beta } > 0$. Management-induced reductions in risk and improvements in reporting that are relatively equal in size would result in null management effects—i.e., $\hat{\beta } \approx 0$. Given the decentralized and largely voluntary nature of civil wildlife strike reporting^34^, we hypothesized that civil-specific wildlife strike metrics were more likely exhibit management-induced reporting biases in the estimated management effects—i.e., $\hat{\beta } \approx 0$ and $\hat{\beta } > 0$. In other words, reporting improvements may offset, or outweigh, the risk-reducing effects of AWHP management. For military-specific wildlife strike metrics, we anticipated that the risk-reducing effects of AWHP management would outweigh any potential management-induced reporting biases due to the more rigorous and consistent reporting standards—i.e., $\hat{\beta } < 0$. Therefore, we expected that estimated management effects derived from military-specific metrics provide a more accurate assessment of the true impact of AWHP management on realized wildlife strike risk, offering a less biased evaluation than those obtained from civil-specific data.

In addition to the static specification given by Eq. 1, which estimates the average management effect over the entire management period, the TWFE regression estimator can also estimate dynamic management effects through an event study specification, as follows:

2 $$\begin{aligned} y_{it} = \beta _{\tau< L} D_{it}^{\tau < L} + \sum _{\tau = L}^{-1} \beta _{\tau } D_{it}^{\tau } + \sum _{\tau =1}^{U} \beta _{\tau } D_{it}^{\tau } + \beta _{\tau> U} D_{it}^{\tau > U} + \theta X_{it} + \alpha _{i} + \delta _{t} + u_{it} \end{aligned}$$

Here, $\tau$ represents the time relative to the AWHP management intervention. In this study, relative time is defined as $\tau \in [-12, 14]$ (Supplementary Information Fig. S1). For instance, an airport that received AWHP management intervention in 2010 would take on relative time values $\tau \in [-5, 11]$, while one receiving intervention in 2016 would take on $\tau \in [-11, 5]$. The $D_{it}^{\tau }$ are dummy variables indicating the presence of AWHP management at airport *i* in year *t*, set to one if it is $\tau$ years from management intervention, and zero otherwise. All other terms in Eq. 2 are defined identically to those in Eq. 1.

The estimated $\beta _{\tau }$ for $\tau \le -1$ are often referred to as treatment *leads* and are commonly used to assess the assumptions of parallel trends and no anticipation, which are essential for the validity of the TWFE regression estimator^45,46^. The estimated $\beta _{\tau }$ for $\tau \ge 1$ are often referred to as treatment *lags* and are interpreted as the average treatment effects on the treated for being exposed to the treatment for $\tau$ periods. Typically, one expects the estimated leads to be statistically indistinguishable from zero, which, in this study, would imply, all else equal, no pre-existing differences in outcomes between non-AWHP and AWHP-managed airports prior to management intervention. Thus, one can be more confident that AWHP management can be implicated when observing estimated lags that significantly deviate from zero. The reference period for the estimated leads and lags in Eq. 2 is the initial year of AWHP intervention, $\tau = 0$. Often, researchers bottom and top code relative time values since the number of sample observations that exist within the tails of the relative time distribution is limited (Supplementary Information Fig. S1). We bottom and top coded relative time values at $L = -8$ and $U = 10$. From Eq. 2, the coefficients associated with the bottom and top coded relative time periods, $\beta _{\tau < L}$ and $\beta _{\tau > U}$, are excluded when reporting our regression results as the estimated coefficients do not have a clear interpretation. Further, given the inherent variability of wildlife strike metrics, we binned relative time periods in two-year intervals so that $\beta _{[-8,-7]}$ captures the estimated impact of AWHP management 7-8 years prior to management intervention and so on, with $\beta _{[9,10]}$ capturing the estimated impact of AWHP management 9-10 years after management intervention.

In our regression models, we incorporated the covariate matrix $X_{it}$ to account for airport-specific, time-variant factors that influence the occurrence of wildlife strikes and their reporting. The control variables included in $X_{it}$ varied depending on the wildlife strike metric being analyzed. For overall and disruptive wildlife strike rates, we controlled for the share of civil aircraft movements that are air carrier or air taxi, since commercial and general aviation have different reporting propensities and aircraft with differing risk profiles^6^. We also controlled for the proportion of total aircraft movements flown by military aircraft, given the dramatic differences in means reported above which are likely driven by differences in risk and reporting across civil and military aviation^15,18^. When estimating the impact of AWHP management on wildlife strike costs, we control for the number damaging wildlife strike incidents that occurred in the same year. Variation in wildlife strike costs, both across and within airports, is determined by variation in both the frequency of cost-inducing wildlife strikes and the severity of such events^2^. By controlling for the number of damaging wildlife strikes in our models, we attempted to isolate on changes in the severity dimension of wildlife strike risk while holding the frequency dimension constant. To document the relative insensitivity of our estimated management effects, we estimated alternative model specifications in the Supplementary Information—including models that excluded covariates.

As mentioned previously, a growing body of econometric methods literature has identified significant limitations with the traditional TWFE regression estimator, demonstrating that it can yield biased results in settings with variation in treatment timing and heterogeneous treatment effects across units^47–51^. To address these concerns, we employ an alternative TWFE estimator that is robust to these pitfalls as recommended by recent methodological advancements^46^. Specifically, we utilize the estimator proposed by Sun and Abraham^49^, which allows for the aggregation of robustly estimated management effects into overall estimates that are analogous to those presented in Eq. 1 and 2. Estimation of Eq. 1 and 2 via Sun and Abraham was performed in R (version 4.3.1) using the package fixest^52,53^.

## Results

### Average management effects

We begin with the average effects of AWHP management—i.e., estimates that reflect average effects across AWHP airports throughout the management period (Table 1). AWHP management was estimated to increase the joint overall wildlife strike rate at the average AWHP-managed airport ($\hat{\beta }=1.24$, $p = 0.002$) (Table 1A). The estimated increase in the joint overall wildlife strike rate appears to have been entirely driven by an increase the civil-specific overall wildlife strike rate ($\hat{\beta }=1.26$, $p = 0.005$) since the estimated management effect on the military-specific rate was negative, though statistically indistinguishable from zero ($\hat{\beta }=-0.95$, $p = 0.708$). Moving from the total number of wildlife strikes, we found no significant impact of AWHP management on the disruptive wildlife strike rate (Table 1B). Again, disruptive wildlife strikes are those that indicated repair costs, non-repair (other) costs, damage, a negative effect on the flight, aircraft downtime, or any combination of these. The estimated management effects across joint ($\hat{\beta } = -0.01$, $p = 0.934$), civil- ($\hat{\beta } = -0.02$, $p = 0.698$), and military-specific ($\hat{\beta } = -1.19$, $p = 0.395$) disruptive strike rates were all negative but exhibit no statistically significant deviation from zero. With respect to wildlife strike costs, AWHP management was estimated to significantly reduce annual joint wildlife strike costs for the average AWHP-managed airport throughout the management period ($\hat{\beta } = -1.12$, $p = 0.031$) (Table 1C). Opposite the estimated management effect on the joint wildlife strike rate, however, which was driven by civil-side effects, the estimated management effect on wildlife strike costs was largely driven by reductions in strike-induced costs to military aircraft ($\hat{\beta } = -0.76$, $p = 0.050$).

**Table 1:** Estimated impact of AWHP management intervention on joint, civil-, and military-specific wildlife strike metrics

	(1)	(2)	(3)
	Joint Metrics	Civil Metrics	Military Metrics
Panel A: Dependent variable is total wildlife strikes per 5,000 movements			
Estimated management effect	$$1.239^{***}$$	$$1.260^{***}$$	-0.946
SE	(0.356)	( 0.407)	(2.495)
95% CI	[0.502, 1.975]	[0.418, 2.101]	[-6.106, 4.215]
Model covariates:			
Airport FE	Yes	Yes	Yes
Year FE	Yes	Yes	Yes
Commercial movement share	Yes	Yes	No
Military movement share	Yes	No	No
Model Obs.	408	408	408
Model $$\hbox {R}^{2}$$	0.830	0.711	0.501
Panel B: Dependent variable is disruptive wildlife strikes per 5,000 movements			
Estimated management effect	-0.009	-0.022	-1.191
SE	(0.104)	(0.063)	(1.373)
95% CI	[-0.223, 0.206]	[-0.140, 0.095]	[-4.030, 1.648]
Model covariates:			
Airport FE	Yes	Yes	Yes
Year FE	Yes	Yes	Yes
Commercial movement share	Yes	Yes	No
Military movement share	Yes	No	No
Model Obs.	408	408	408
Model $$\hbox {R}^{2}$$	0.637	0.509	0.452
Panel C: Dependent variable is wildlife strike costs (Millions, 2023 $)			
Estimated management effect	-1.116**	-0.366	-0.763*
SE	(0.485)	(0.332)	(0.369)
95% CI	[-2.120, -0.112]	[-1.053, 0.322]	[-1.527, 0.001]
Model covariates:			
Airport FE	Yes	Yes	Yes
Year FE	Yes	Yes	Yes
Civil damaging strikes	Yes	Yes	No
Military damaging strikes	Yes	No	Yes
Model Obs.	408	408	408
Model $$\hbox {R}^{2}$$	0.219	0.291	0.208

NOTE: *Clustered (airport) standard errors. Estimates that satisfy traditional levels of statistical significance indicated by * * *p* $< 0.1$, ** *p* $< 0.05$, *and* *** *p* $< 0.01$

**Fig. 3:**
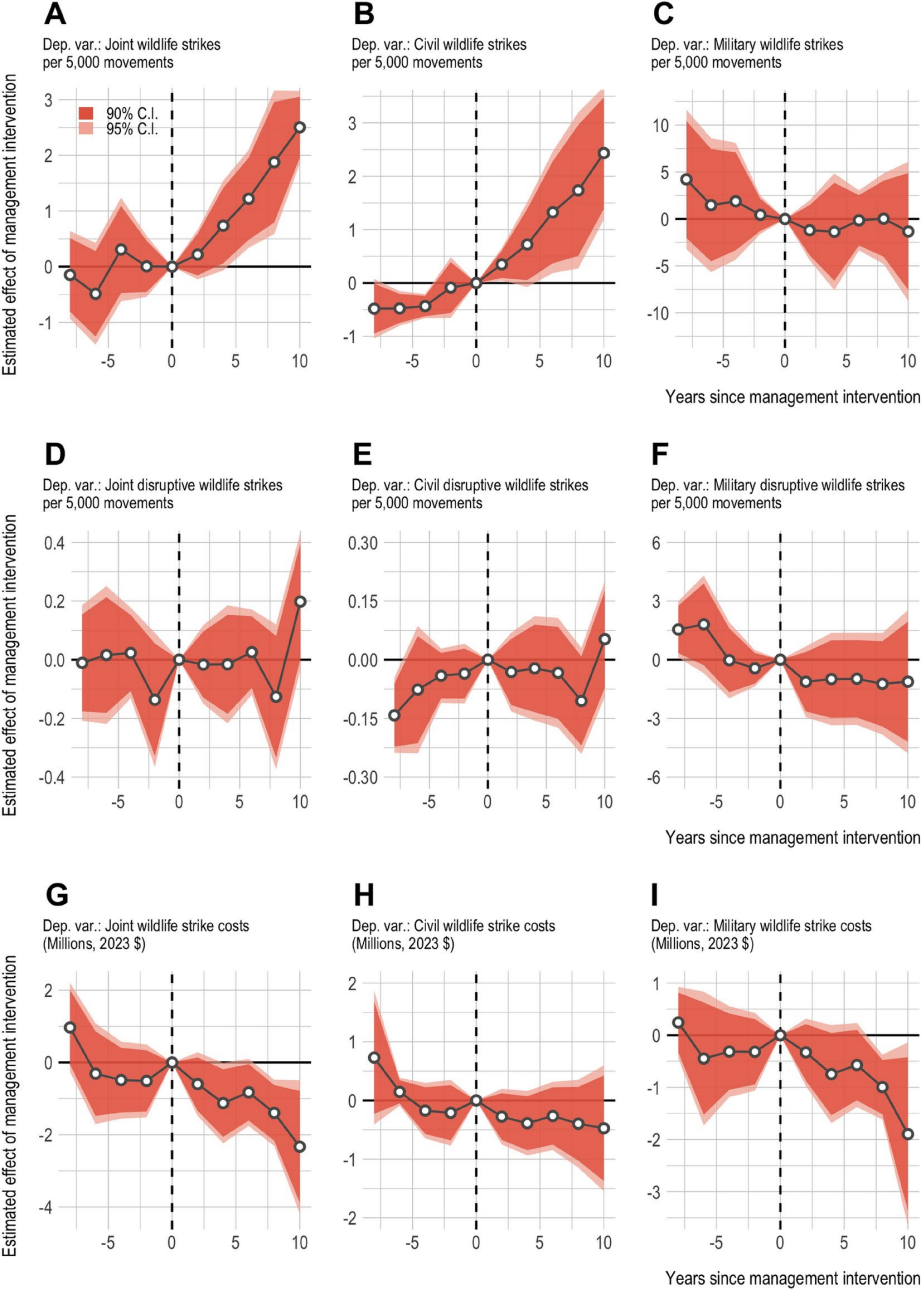
Estimated change in joint, civil, and military wildlife strike metrics before and after management intervention.

### Dynamic management effects

Next we present the dynamic effects AWHP management—i.e., estimates that reflect average effects across AWHP airports at different points in the management period (Fig. 3 & Supplementary Information Table S3). AWHP management was estimated to have an increasingly positive impact on the joint wildlife strike rate over the management period (Fig. 3A). The smallest estimated management effect was observed in the first two years following management intervention ($\hat{\beta }_{[1,2]}=0.22$, $p = 0.347$) with steadily increasing management effects observed over the management period ($\hat{\beta }_{[5,6]}=1.22$, $p = 0.012$; $\hat{\beta }_{[9,10]}=2.50$, $p < 0.000$). The estimated management effects observed in the pre-management period were effectively null, failing tests of statistical significance at traditional levels of confidence both individually and collectively. Well-estimated null effects in the pre-management period, when AWHP management was not present, supplies confidence that our regression models were able to robustly identify the the impact of AWHP management in the post-intervention period. Similar to the static estimates presented above, a comparison of the dynamic management effects among civil- and military-specific wildlife strike rates shows that management-induced changes in the joint wildlife strike rate were again driven by changes in reported wildlife strike incidents with civil aircraft ($\hat{\beta }_{[1,2]}=0.35$, $p = 0.035$; $\hat{\beta }_{[5,6]}=1.33$, $p = 0.032$; $\hat{\beta }_{[9,10]}=2.44$, $p = 0.001$) (Fig. 3B). Among the pre-management estimates for the civil-specific overall wildlife strike rate, there appeared to be some evidence of anticipation in the two years prior to AWHP intervention. The estimated dynamic impact of management on the military wildlife strike rate was generally negative but statistically insignificant across all pre- and post-management intervention periods (Figure ($\hat{\beta }_{[1,2]}=-1.20$, $p = 0.469$; $\hat{\beta }_{[5,6]}=-0.15$, $p = 0.928$; $\hat{\beta }_{[9,10]}=-1.33$, $p = 0.729$) (Fig. 3C).

Similar to the average management effects presented in the previous section, the dynamic impact of AWHP management on joint, civil-, and military-specific disruptive wildlife strike rates exhibited no statistically significant deviation from zero (Fig. 3D–3F). With respect to the military-specific disruptive wildlife strike rate, all estimated management effects were negative throughout the management period but all fail tests of significance at traditional levels of statistical confidence ($\hat{\beta }_{[1,2]}=-1.13$, $p = 0.235$; $\hat{\beta }_{[5,6]}=-0.98$, $p = 0.425$; $\hat{\beta }_{[9,10]}=-1.12$, $p = 0.553$) (Fig. 3F).

AWHP management was estimated to increasingly reduce joint wildlife strike costs throughout the management period (Figure 3G). The largest estimated management effects were observed late in the management period ($\hat{\beta }_{[1,2]}=-0.598$, $p = 0.196$; $\hat{\beta }_{[5,6]}=-0.825$, $p = 0.095$; $\hat{\beta }_{[9,10]}=-2.33$, $p = 0.021$). For both civil- and military-specific wildlife strike costs, the estimated dynamic impact of AWHP management was increasingly negative, generally, over the management period. The estimated dynamic impact of AWHP management on civil wildlife strike costs was increasingly negative but statistically indistinguishable from zero across the management period ($\hat{\beta }_{[1,2]}=-0.28$, $p = 0.266$; $\hat{\beta }_{[5,6]}=-0.27$, $p = 0.382$; $\hat{\beta }_{[9,10]}=-0.47$, $p = 0.394$) (Fig. 3H). AWHP management was estimated to reduce military wildlife strike costs at the average AWHP-managed airport throughout the management period, with the largest and most statistically robust effects being observed later in the management period ($\hat{\beta }_{[1,2]}=-0.33$, $p = 0.328$; $\hat{\beta }_{[5,6]}=-0.57$, $p = 0.178$; $\hat{\beta }_{[9,10]}=-1.90$, $p = 0.046$) (Fig. 3I). The estimated dynamic management effects reiterate the estimated average management effects, that AWHP management was associated with an increase in the frequency of wildlife strike incidents with civil aircraft and a decrease in joint wildlife strike costs among AWHP-managed airports over the management period, with the latter being largely driven by a reduction in strike-induced cost to military aircraft. However, the dynamic management effects indicate the estimated impact of AWHP management intervention grew with tenure.

**Table 2:** Heterogeneity in the estimated impact of wildlife hazards management intervention when partitioning managed airports into low versus high management presence (staff years employed).

	(1)	(2)	(3)
	Joint Metrics	Civil Metrics	Military Metrics
Panel A: Dependent variable is total wildlife strikes per 5,000 movements^a^			
Estimated *low*-management effect	$$0.902^{***}$$	$$0.608^{***}$$	2.090
SE	(0.198)	(0.155)	(1.337)
95% CI	[0.484, 1.319]	[0.28, 0.936]	[-0.731, 4.911]
Model Obs.	306	306	306
Model $$\hbox {R}^{2}$$	0.851	0.742	0.440
Estimated *high*-management effect	$$1.580^{**}$$	$$1.913^{**}$$	-4.440**
SE	(0.583)	(0.716)	(2.060)
95% CI	[0.344, 2.816]	[0.395, 3.431]	[-8.807, -0.073]
Model Obs.	289	289	289
Model $$\hbox {R}^{2}$$	0.831	0.728	0.618
Panel B: Dependent variable is disruptive wildlife strikes per 5,000 movements^b^			
Estimated *low*-management effect	-0.046	0.032	-0.334
SE	(0.080)	(0.036)	(0.400)
95% CI	[-0.214, 0.122]	[-0.043, 0.108]	[-1.178, 0.511]
Model Obs.	306	306	306
Model $$\hbox {R}^{2}$$	0.626	0.519	0.359
Estimated *high*-management effect	-0.024	-0.097	-2.253***
SE	(0.078)	(0.111)	(0.407)
95% CI	[-0.189, 0.141]	[-0.332, 0.138]	[-3.116, -1.39]
Model Obs.	289	289	289
Model $$\hbox {R}^{2}$$	0.660	0.511	0.579
Panel C: Dependent variable is wildlife strike costs (Millions, 2023 $)^c^			
Estimated *low*-management effect	-0.250	-0.121	-0.16
SE	(0.235)	(0.078)	(0.253)
95% CI	[-0.747, 0.246]	[-0.285, 0.044]	[-0.694, 0.373]
Model Obs.	306	306	306
Model $$\hbox {R}^{2}$$	0.230	0.204	0.233
Estimated *high*-management effect	-2.006***	-0.593***	-1.409**
SE	(0.600)	( 0.200)	(0.625)
95% CI	[-3.278, -0.733]	[-1.017, -0.17]	[-2.733, -0.084]
Model Obs.	289	289	289
Model $$\hbox {R}^{2}$$	0.236	0.375	0.214

NOTES: *Clustered (airport) standard errors. Estimates that satisfy traditional levels of statistical significance indicated by* * *p* $< 0.1$, ** *p* $< 0.05$, *and* *** *p* $< 0.01$. ^a^ *See the columns of Panel A Table* 1* for model covariates.* ^b^ *See the columns of Panel B Table* 1* for model covariates.* ^c^ *See the columns of Panel C Table* 1* for model covariates *

### Heterogeneous management effects

In our last set of analyses, we investigated how the estimated average management effects behave when partitioning the sample of AWHP airports into two groups: those with relatively low and high levels of AWHP management staffing over the management period. Those airports categorized as low-management airports ($n = 7$) averaged approximately 0.75 AWHP staff years per year (SD = 0.376) throughout the management period, while those categorized as high-management airports ($n = 6$) had an average 1.21 AWHP staff years per year (SD = 0.346) throughout the management period (see Supplementary Information Fig. S2). If the AWHP is to be implicated as main cause of the estimated management effects observed in the previous two sections, we expected the estimated management effects to be pronounced among high-management airports^54^. Further, recall that non-AWHP airports were not necessarily unmanaged airports. Investigating the heterogeneous impact of management intervention *among* AWHP-managed allowed us to estimate the impact of airport wildlife hazards management more generally.

The estimated impact of AWHP management on the joint wildlife strike rate was positive and statistically significant across both low- and high-management airports (Table 2A). As expected, however, the counterintuitive increase in the joint overall wildlife strike rate was larger among high-management ($\hat{\beta } = 1.58$, $p = 0.015$) relative to low-management airports ($\hat{\beta } = 0.90$, $p < 0.000$). The heterogeneous impact of AWHP management on the civil wildlife strike rate was even more pronounced, with the estimated positive impact of AWHP management among high-management airports ($\hat{\beta } = 1.91$, $p = 0.017$) being roughly 3x larger than that estimated for low-management airports ($\hat{\beta } = 0.61$, $p = 0.001$). Oppositely, the estimated management effect on the military wildlife strike rate was estimated to be positive and statistically insignificant among low-management airports ($\hat{\beta } = 2.09$, $p = 0.136$), but robustly negative among high-management airports ($\hat{\beta } = -4.44$, $p = 0.047$). The estimated impact of AWHP management on the disruptive wildlife strike rate remained effectively null, as was the case in previously discussed results (Table 2B). The one exception, however, was the estimated management effect among high-management airports that suggested a meaningful decrease in military-specific disruptive wildlife strike rate ($\hat{\beta } = -2.25$, $p < 0.000$). Lastly, the estimated impact of AWHP management on joint wildlife strike costs was significantly larger in absolute magnitude among high-management ($\hat{\beta } = -2.01$, $p = 0.004$) relative to low-management airports ($\hat{\beta } = -0.25$, $p = 0.303$) (Table 2C). Further, AWHP management was estimated to reduce both civil- ($\hat{\beta } = -0.59$, $p = 0.009$) and military-specific ($\hat{\beta } = -1.41$, $p = 0.039$) wildlife strike costs among high-management airports throughout the management period, though management-induced reductions in joint wildlife strike costs continued to be driven by management-induced reductions military wildlife strike costs. These results demonstrated that the estimated average management effects presented in Table 1 were largely driven by management effects among high-management airports. Additionally, where we anticipated reporting to be most robust—i.e., military metrics—and where management is most pronounced—i.e., high-management airports—the estimated effects of AWHP management on the overall wildlife strike rate ($\hat{\beta } = -4.44$, $p = 0.047$), the disruptive wildlife strike rate ($\hat{\beta } = -2.25$, $p < 0.000$), and wildlife strike costs ($\hat{\beta } = -1.41$, $p = 0.039$) all reflected a reduction in realized wildlife strike risk.

### Sensitivity analyses

In the attached Supplementary Information, we conducted several ancillary analyses to test the robustness of our primary findings. First, we address the components driving changes in wildlife strike rates by estimating management effects using counts of wildlife strikes instead of rates, as detailed in Supplementary Information Table S3. In Supplementary Information Table S4 we provided an analog to our main results presented in Table 1 where we re-estimated our regression models under alternative specifications. Specifically, we present how the estimated management effects behaved when we excluded covariates from our regression models as well as when we included flyway-by-year fixed effects to account for differential time trends and shocks that may be due to regional differences in bird migrations and populations, among other factors. Next, since the information reported in wildlife strike records is sometimes incomplete, it is often difficult to perfectly identify which wildlife strike incidents occurred within the airport operations area (AOA) and which occurred at some distance from the airport. If AWHP management is to be implicated in the results presented above, one would expect that the estimated management effects were largely due to changes in near- or on-airport wildlife strike incidents. We reduced the number of wildlife strike records included in our sample to only include those which could be *confirmed* within the AOA and reestimated our Table 1 average management effects, as presented in Supplementary Information Table S5. Finally, there were three airports in our sample that can be considered outliers in terms of their operational size and characteristics; Portland International Airport (PDX), Phoenix International Airport (PHX), and Salt Lake City International Airport (SLC). In Supplementary Information Table S6 we, again, reestimated our Table 1 average management effects when these airports were excluded from the sample. While the magnitude and precision of the estimated management effects vary, the relative stability of these estimates across various model specifications and sample definitions supplied confidence in the robustness of our main findings, especially given our limited sample size.

## Discussion

The straightforward evaluation of wildlife hazards management at airports is undermined by the latency of underlying wildlife strike risk and imperfect reporting systems that bias measures of realized wildlife strike risk. As a result, many have opted for evaluating wildlife hazards management through case studies of species-specific management strategies and species-specific outcomes^28–33^. Though indispensable, these studies often fail to provide the comprehensive program evaluation that is useful to airport managers and policymakers^27^. The goal of this study was to fill this gap by (1) focusing on aggregate measures of wildlife strike risk and (2) employing a strategic research design that allowed for the decoupling of management-induced reporting and risk effects. Our study found that airport wildlife hazards management, as measured through AWHP management intervention, has both a positive impact on wildlife strike reporting and negative impact on realized wildlife strike risk.

Specifically, our estimates indicate that AWHP management was associated with an increase in the frequency of wildlife strike incidents among AWHP-managed airports. This result, however, was entirely driven by a management-induced increase in civil wildlife strike incidents while there was no parallel management-induced increase in the overall wildlife strike rate among military aircraft. As anticipated by our conceptual framework, the heterogeneous impact of AWHP management on civil- and military-specific wildlife strike rates suggests management-induced changes in civil wildlife strike reporting as opposed to changes in wildlife strike risk. In other words, AWHP management intervention dramatically increased the frequency of *reported* wildlife strike incidents with civil aircraft. The impact of AWHP management, and airport wildlife hazards management generally, on wildlife strike reporting is itself valuable. To effectively and efficiently manage wildlife hazards, airport and wildlife managers must first have a proper understanding of wildlife strike risk at their airport^17,27^. While other sources of data can be utilized–e.g., point count surveys—more complete and representative wildlife strike records can only aid in the formulation and execution of effective wildlife hazards management plans^8–11^. Further, improvements in wildlife strike reporting have benefits beyond airport-specific management strategies, enriching the science of wildlife strike risk generally^27,55^.

We consistently estimated that AWHP management was associated with a decrease in wildlife strike costs, indicating a management-induced reduction in realized wildlife strike risk. The estimated average impact of AWHP management on joint wildlife strike costs delivers an overall cost savings of $147.3 million among AWHP-managed airports from 2008 to 2021, suggesting large returns to robust airport wildlife hazards management. Specifically, the total funding required by the AWHP among managed sample airports was roughly $19.4 million from 2008 to 2021, or roughly $0.15 million per full management year. The estimated reduction in joint wildlife strike costs over this same period suggests that the benefits of such a management program exceed the costs by a ratio of 7:1. Moreover, the savings implied by the military-specific cost estimates alone—which were estimated to be $100.7 million from 2008 to 2021—deliver a benefit-cost ratio of 5:1. It is important to note however, that the estimated benefits of AWHP management, or airport wildlife hazards management generally, were not evenly distributed throughout the management period and across AWHP-managed airports. Specifically, the estimated cost-reducing benefits of AWHP management grew over the management period. This emphasizes the importance of long-term management plans that build on historical knowledge and practice, or large-scale management strategies that are executed over multi-year periods such as habitat modifications or prescribed land-use changes^16,56^. Additionally, the estimated cost-reducing benefits of AWHP management were concentrated among high-management airports, all of which employed > 1 AWHP staff year, on average, throughout the management period. This implies that, in order to realize the full benefits of airport wildlife hazards management, airports should be careful to invest in sufficient management effort and staffing.

Given that disruptive wildlife strikes are inclusive of strike records that report costs, and given the estimated impact of AWHP management on strike-induced costs, it was surprising that we found no impact of AWHP management on the joint, civil-, or military-specific disruptive wildlife strike rate. The estimated impact of AWHP was consistently negative, but not significant at traditional levels of statistical confidence. Disruptive wildlife strike events are rare—the average airport in our sample recorded roughly 4 per year. The scarcity of such incidents, combined with our restricted sample of 24 airports over 17 years, likely generated a lack of statistical power. Relatedly, our limited sample, which was restricted by data availability and the specific requirements of our research design, presents a significant limitation to our study. While we are confident that our estimates accurately reflect the impact of AWHP management within our sample of airports, it is important to note that generalizing these estimates to a broader population may not be appropriate. An additional limitation is that our study does not compare unmanaged and managed airports, generally. While such a research design is preferable, it was infeasible due to FAA regulations^9^. As a result, the non-AWHP airports in our sample were likely to have some level of wildlife hazards assessment and management. Unfortunately, the lack of management documentation at non-AWHP airports prevented us from measuring the timing and intensity of their potential management activities. Therefore, our study compared airports that adopted AWHP management to those that did not and our results should be interpreted with this in mind.

The challenge of evaluating airport wildlife hazards management programs, through their simultaneous impacts on risk and reporting, is not unique to the wildlife strike phenomenon. Such a characterization is likely applicable across many domains of risk and wildlife management, where the measurement of realized risk often becomes more complete in the presence of management due to increased attention and a desire to evaluate program effectiveness. Our study contributes to a growing body of literature that demonstrates the leveraging of observational data and strategic research designs to evaluate the causal impact and value of such management programs^40–43,54,57^. Further, airport wildlife hazards management, similar to the management of other infrequent but high risk phenomenon such as forest fires and disease outbreaks, suffers the problem of attribution. Often, when these management programs are effective, rare events become more infrequent—e.g., no severe wildlife strike incidents, no forest fires, no disease outbreaks—which undermines the impetus for management^58^. Evidence-based program evaluation, as exemplified here and in other studies, helps to prevent the misattribution of outcomes by clearly delineating the role of management, particularly in situations where risk is characterized by infrequent but high-consequence incidents^43,54,57^. The results of our study, documenting the role of the AWHP in improving wildlife strike reporting and reducing realized wildlife strike risk, provides empirical evidence that airport wildlife hazards management can be both effective and economically viable. Such results are useful to airport managers and policymakers who are concerned with the efficient use of resources to mitigate wildlife strike risk and protect the flying public^1^. Moreover, the increasing prevalence of human-wildlife conflict, coupled with growing concerns over biodiversity loss, presents a significant challenge for wildlife managers and policymakers globally^59–61^. To address these challenges effectively, evidence-based approaches and evaluations are essential for designing sustainable and adequate wildlife management plans^17,62^.

## Supplementary Information

Supplementary Information.

## Data Availability

The datasets generated and analyzed during the current study are available from the corresponding author on reasonable request.
