# Supplementary material for: Estimating the impact of airport wildlife hazards management on realized wildlife strike risk
Source: Sci Rep. 2024 Nov 22;14:29018. doi: 10.1038/s41598-024-79946-3 (PMC11584785; doi:10.1038/s41598-024-79946-3)
Supplement: Supplementary file 1 — Supplementary Information. [file 41598_2024_79946_MOESM1_ESM.pdf]

Supplementary Information

Manuscript:
Estimating the impact of airport wildlife hazards management on realized economic risk

Authors:
Levi Altringer[a+], Michael J. Begier[b], Jenny E. Washburn[c], and Stephanie A. Shwiff[a]

Affiliations:
US Department of Agriculture, Animal and Plant Health Inspection Service, Wildlife Services, National Wildlife Research Center, Fort Collins, CO, 80521, USA
US Department of Agriculture, Animal and Plant Health Inspection Service, Wildlife Services, Airport Wildlife Hazards Program, Washington, DC, 20250, USA
US Department of Agriculture, Animal and Plant Health Inspection Service, Wildlife Services, Airport Wildlife Hazards Program, Sandusky, OH, 44870 , USA

[+]Corresponding author. Email: Levi.Altringer@usda.gov

Table of Contents

Supplementary Table S1. Data summary statistics……………..…………………..……1

Supplementary Table S2. Estimated dynamic effects of wildlife hazards management on join, civil-, and military-specific wildlife strike metrics (estimates presented visually in Figure 3 of manuscript)……………………….…………...….2

Supplementary Table S3. Estimated impact of management intervention on wildlife strike counts a la Wooldridge (2022)………………………..…………………4

Supplementary Table S4. Sensitivity of estimated ATTs to alternative model specifications…………………………………………………………………….…5

Supplementary Table S5. Sensitivity of estimated ATTs when subsetting to only those wildlife strikes that are confirmed within the airport operations area (AOA)…6

Supplementary Table S6. Sensitivity of estimated ATTs to the exclusion of PDX, PHX, and SLC……………………..……………………………………………….7

Supplementary Figure S1. Distribution of pre-management and management observations across time relative to AWHP management intervention…………...8

Supplementary Figure S2. Average number of staff years funded over relative time at those airports with below- (low) and above-median (high) Airport Wildlife Hazards Program staff years throughout the management period...............….…9

Table S1. Data summary statistics

Sample	
Airports	24
Non-treated	11
Treated	13
Years (2005-2021)	17
Total obs.	408

Summary Statistics	Mean	SD	Min	Max
Aircraft movements				
Joint	96,584	105,539	9,502	563,536
Civil	88,645	106,854	4,701	560,561
Military	7,829	6,348	277	34,992
Air carrier share of civil movements	0.35	0.28	0.00	0.96
Military share of joint movements	0.16	0.16	0.00	0.75
Total wildlife strikes				
Joint	29.17	39.35	0.00	280.00
Civil	23.01	38.65	0.00	266.00
Military	6.15	7.75	0.00	46.00
Total wildlife strikes per 5,000 movements				
Joint	1.87	2.01	0.00	11.76
Civil	1.41	1.80	0.00	15.83
Military	5.30	7.58	0.00	62.65
Disruptive wildlife strikes				
Joint	3.99	4.77	0.00	27.00
Civil	2.22	3.82	0.00	22.00
Military	1.78	3.03	0.00	20.00
Disruptive wildlife strikes per 5,000 movements				
Joint	0.32	0.46	0.00	2.53
Civil	0.13	0.16	0.00	16.87
Military	1.42	2.64	0.00	
Damaging wildlife strikes				
Joint	1.86	2.87	0.00	16.00
Civil	1.18	2.64	0.00	15.00
Military	0.69	1.30	0.00	8.00
Wildlife strike costs (Millions, 2023 $)				
Joint	0.39	1.89	0.00	28.29
Civil	0.15	0.79	0.00	7.95
Military	0.24	1.66	0.00	28.29

SOURCES: *Civil and military aircraft movement data come from the FAA's Air Traffic Activity Data System (publicly available). Civil wildlife strike data come from reported incidents in the FAA's National Wildlife Strike Database (publicly available). Military (Air National Guard) wildlife strike data come from reported incidents in the Air Force Safety Automated System (restricted access). Wildlife hazards management program data come from the USDA-APHIS-WS Airport Wildlife Hazards Program (restricted access).*

Table S2. Estimated dynamic effects of wildlife hazards management on join, civil-, and military-specific wildlife strike metrics (estimates presented visually in Figure 3 of manuscript).

	(1) Joint Metrics		(2) Civil Metrics		(3) Military Metrics	
Time (years) relative to management intervention:	ATT	SE	ATT	SE	ATT	SE
Panel A: Dependent variable is total wildlife strikes per 5,000 movements						
[-8, -7]	-0.148	(0.402)	-0.481	(0.284)	4.216	(3.790)
[-6, -5]	-0.487	(0.466)	-0.476***	(0.165)	1.474	(3.641)
[-4, -3]	0.308	(0.475)	-0.432***	(0.115)	1.887	(3.171)
[-2, -1]	0.006	(0.279)	-0.086	(0.290)	0.439	(1.041)
0			*(Reference period)*			
[1, 2]	0.218	(0.227)	0.349**	(0.155)	-1.197	(1.627)
[3, 4]	0.736*	(0.411)	0.722*	(0.406)	-1.363	(3.168)
[5, 6]	1.219**	(0.448)	1.326**	(0.581)	-0.148	(1.628)
[7, 8]	1.876***	(0.658)	1.735**	(0.746)	0.029	(2.461)
[9, 10]	2.503***	(0.334)	2.435***	(0.634)	-1.330	(3.786)
Model covariates:						
Airport FE	Yes		Yes		Yes	
Year FE	Yes		Yes		Yes	
Carrier movement share	Yes		Yes		No	
Military movement share	Yes		No		No	
Model Obs.	408		408		408	
Model R^2	0.830		0.711		0.501	
Panel B: Dependent variable is disruptive wildlife strikes per 5,000 movements						
[-8, -7]	-0.011	(0.101)	-0.142***	(0.049)	1.547**	(0.736)
[-6, -5]	0.016	(0.120)	-0.077	(0.083)	1.806	(1.272)
[-4, -3]	0.023	(0.079)	-0.041	(0.035)	-0.029	(0.989)
[-2, -1]	-0.137	(0.117)	-0.035	(0.039)	-0.439	(0.474)
0			*(Reference period)*			
[1, 2]	-0.017	(0.069)	-0.031	(0.051)	-1.126	(0.923)
[3, 4]	-0.016	(0.103)	-0.022	(0.068)	-0.994	(1.204)
[5, 6]	0.026	(0.074)	-0.033	(0.072)	-0.977	(1.202)
[7, 8]	-0.126	(0.126)	-0.105	(0.069)	-1.235	(1.324)
[9, 10]	0.198	(0.119)	0.052	(0.075)	-1.124	(1.867)
Model covariates:						
Airport FE	Yes		Yes		Yes	
Year FE	Yes		Yes		Yes	
Carrier movement share	Yes		Yes		No	
Military movement share	Yes		No		No	
Model Obs.	408		408		408	
Model R^2	0.637		0.509		0.452	

Table continued on next page.

Table continued.

Panel C: Dependent variable is wildlife strike costs (Millions, 2023 $)

[-8, -7]	0.969	(0.627)	0.728	(0.581)	0.243	(0.349)
[-6, -5]	-0.309	(0.711)	0.146	(0.121)	-0.448	(0.654)
[-4, -3]	-0.487	(0.544)	-0.173	(0.242)	-0.315	(0.444)
[-2, -1]	-0.511	(0.514)	-0.211	(0.286)	-0.319	(0.381)
0			*(Reference period)*			
[1, 2]	-0.598	(0.449)	-0.276	(0.242)	-0.329	(0.329)
[3, 4]	-1.124*	(0.568)	-0.389	(0.280)	-0.746	(0.479)
[5, 6]	-0.825*	(0.475)	-0.265	(0.297)	-0.572	(0.411)
[7, 8]	-1.395***	(0.468)	-0.397	(0.382)	-0.994***	(0.317)
[9, 10]	-2.332**	(0.940)	-0.474	(0.546)	-1.896**	(0.897)
Model covariates:						
Airport FE	Yes		Yes		Yes	
Year FE	Yes		Yes		Yes	
Civil damaging strikes	Yes		Yes		No	
Military damaging strikes	Yes		No		Yes	
Model Obs.	408		408		408	
Model R^2	0.219		0.291		0.208	

NOTES: *Clustered (airport) standard errors. Estimates that satisfy traditional levels of statistical significance indicated by * p < 0.1, ** p < 0.05, and *** p < 0.01. All models are estimated a la Sun & Abraham (2021).*

Table S2. Estimated impact of management intervention on wildlife strike counts a la Wooldridge (2022)

	(1)<br>Joint Metrics	(2)<br>Civil Metrics	(3)<br>Military Metrics
Panel A: Dependent variable is count of all wildlife strikes			
ATT	13.057*	12.880**	1.478
SE	(6.994)	(6.259)	(2.879)
Panel B: Dependent variable is count of disruptive wildlife strikes			
ATT	-0.746	-0.200	-0.371
SE	(0.738)	(0.517)	(0.877)

NOTES: *Each cell presents the estimated ATT from a distinct regression model estimated a la Wooldridge (2022) with no covariates included. Clustered (airport) standard errors. Estimates that satisfy traditional levels of statistical significance indicated by * $p < 0.1$, ** $p < 0.05$, and *** $p < 0.01$.*

Table S4. Sensitivity of estimated ATTs to alternative model specifications

Model specifications:					(1)	(2)	(3)
Airport FEs	Year FEs	Flyway-Year FEs	Additional Covariates		Joint Metrics	Civil Metrics	Military Metrics
Panel A: Dependent variable is total wildlife strikes per 5,000 movements							
Yes	Yes	No	No	ATT<br>SE	1.320***<br>(0.365)	1.200**<br>(0.415)	-0.946<br>(2.495)
Yes	Yes	No	Yes	ATT<br>SE	1.239***<br>(0.356)	1.260***<br>(0.407)	
Yes	No	Yes	Yes	ATT<br>SE	1.338***<br>(0.439)	1.340***<br>(0.426)	-0.461<br>(3.342)
Panel B: Dependent variable is disruptive wildlife strikes per 5,000 movements							
Yes	Yes	No	No	ATT<br>SE	-0.001<br>(0.108)	-0.027<br>(0.056)	-1.191<br>(1.373)
Yes	Yes	No	Yes	ATT<br>SE	-0.009<br>(0.104)	-0.022<br>(0.057)	
Yes	No	Yes	Yes	ATT<br>SE	0.025<br>(0.125)	-0.03<br>(0.050)	-1.17<br>(1.474)
Panel C: Dependent variable is wildlife strike costs (Millions, 2023 $)							
Yes	Yes	No	No	ATT<br>SE	-0.969*<br>(0.506)	-0.361<br>(0.325)	-0.608<br>(0.384)
Yes	Yes	No	Yes	ATT<br>SE	-1.116**<br>(0.485)	-0.366<br>(0.332)	-0.763*<br>(0.369)
Yes	No	Yes	Yes	ATT<br>SE	-1.127**<br>(0.449)	-0.429<br>(0.326)	-0.727**<br>(0.319)

NOTES: *Each cell presents the estimated ATT from a distinct regression model. Clustered (airport) standard errors. Estimates that satisfy traditional levels of statistical significance indicated by * p < 0.1, ** p < 0.05, and *** p < 0.01. All models are estimated a la Sun & Abraham (2021). Highlighted cells correspond to those reported in Table 1 of the manuscript (also Table S2 above).*

Table S5. Sensitivity of estimated ATTs when subsetting to only those wildlife strikes that are confirmed within the airport operations area (AOA)

	(1)<br>Joint Metrics	(2)<br>Civil Metrics	(3)<br>Military Metrics
Panel A: Dependent variable is total wildlife strikes per 5,000 movements			
All reported strikes included:			
ATT	1.239***	1.260***	-0.946
SE	(0.356)	(0.407)	(2.495)
Only AOA-confirmed strikes included:			
ATT	1.131***	1.177***	-1.041
SE	(311)	(0.351)	(1.532)
Panel B: Dependent variable is disruptive wildlife strikes per 5,000 movements			
All reported strikes included:			
ATT	-0.009	-0.022	-1.191
SE	(0.104)	(0.057)	(1.373)
Only AOA-confirmed strikes included:			
ATT	0.077**	-0.010	-0.604
SE	(0.035)	(0.034)	(0.818)
Panel C: Dependent variable is wildlife strike costs (Millions, 2023 $)			
All reported strikes included:			
ATT	-1.116**	-0.366	-0.763*
SE	(0.485)	(0.332)	(0.369)
Only AOA-confirmed strikes included:			
ATT	-1.011*	-0.442	-0.579
SE	(0.538)	(0.416)	(0.391)

NOTES: *Each cell presents the estimated ATT from a distinct regression model. Clustered (airport) standard errors in parentheses. Estimates that satisfy traditional levels of statistical significance indicated by * p < 0.1, ** p < 0.05, and *** p < 0.01. All models are estimated a la Sun & Abraham (2021). Highlighted cells correspond to those reported in Table 1 of the manuscript. All models include the covariates listed the respective panels and columns of Table 1 in the manuscript (also Table S2 above).*

Table S6. Sensitivity of estimated ATTs to the exclusion of PDX, PHX, and SLC

	(1)<br>Joint Metrics	(2)<br>Civil Metrics	(3)<br>Military Metrics
Panel A: Dependent variable is total wildlife strikes per 5,000 movements			
All sample airports included:			
ATT	1.239***	1.260***	-0.946
SE	(0.356)	(0.407)	(2.495)
PDX, PHX, and SLC excluded:			
ATT	1.220**	1.231**	1.616
SE	(0.396)	(0.453)	(1.391)
Panel B: Dependent variable is disruptive wildlife strikes per 5,000 movements			
All reported strikes included:			
ATT	-0.009	-0.022	-1.191
SE	(0.104)	(0.057)	(1.373)
PDX, PHX, and SLC excluded:			
ATT	0.002	-0.030	0.335
SE	(0.118)	(0.064)	(0.541)
Panel C: Dependent variable is wildlife strike costs (Millions, 2023 $)			
All reported strikes included:			
ATT	-1.116**	-0.366	-0.763*
SE	(0.485)	(0.332)	(0.369)
PDX, PHX, and SLC excluded:			
ATT	-0.866**	0.034	-0.900*
SE	(0.408)	(0.109)	(0.432)

NOTES: *Each cell presents the estimated ATT from a distinct regression model. Clustered (airport) standard errors in parentheses. Estimates that satisfy traditional levels of statistical significance indicated by * p < 0.1, ** p < 0.05, and *** p < 0.01. All models are estimated a la Sun & Abraham (2021). Highlighted cells correspond to those reported in Table 1 of the manuscript. All models include the covariates listed their respective panels and columns of Table 1 in the manuscript (also Table S2 above).*

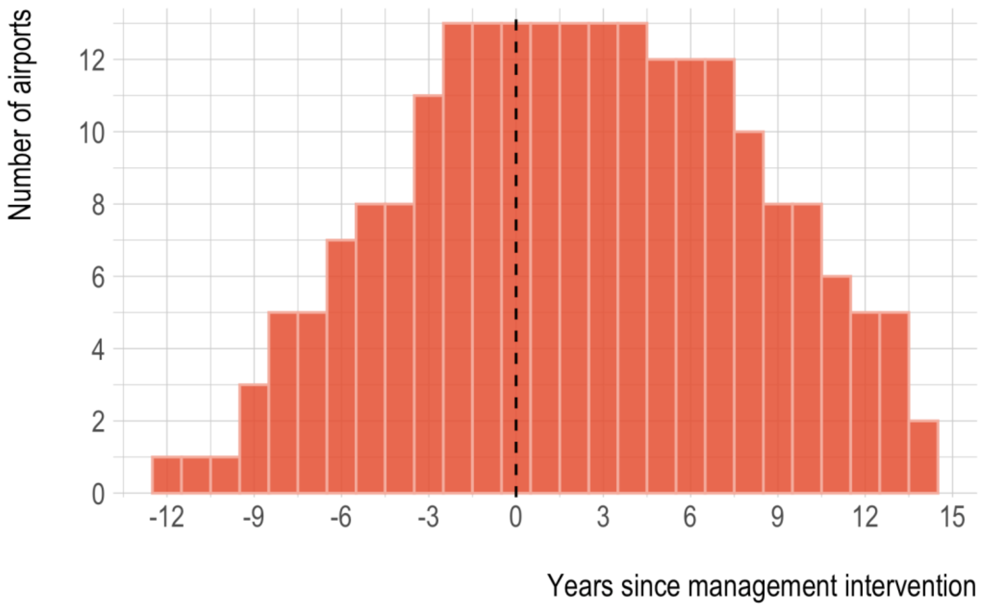

Figure S1. Distribution of pre-management and management observations across time relative to AWHP management intervention. In our analysis, relative time values are bottom- and top-coded at *-8* and *10*, respectively. Further, for relative time values *[-8, … , -1]* and *[1, … , 10]*, we bin adjacent periods (e.g., *[-8, -7]*, *[-6, -5]*, up to *[9, 10]*) with the initial year of management intervention (i.e., relative time period 0) being defined as the reference period.

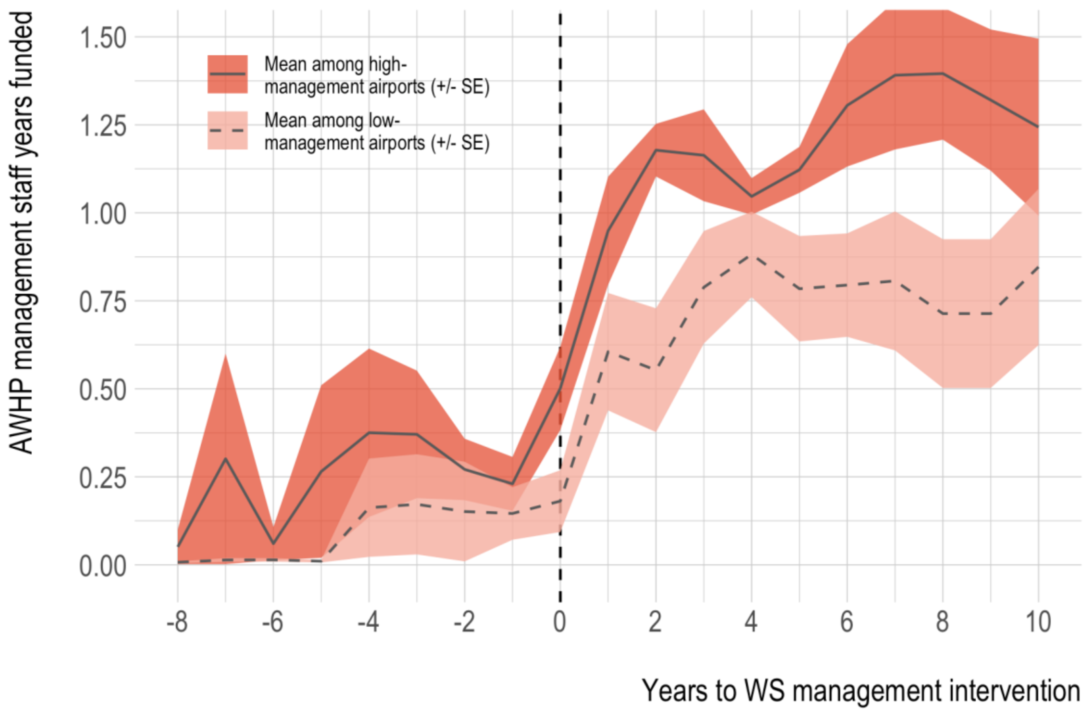

Figure S2. Average number of staff years funded over relative time at those airports with below- (low) and above-median (high) Airport Wildlife Hazards Program staff years throughout the management period. The airports categorized as low-management airports are BAF, BHM, FOE, GTF, LMT, MTN, and STJ. This group of airports experienced an average 0.748 funded staff years in the management period. Airports categorized as high-management airports are BTV, FWA, PIA, SLC, SUX, and SWF. This group of airports experienced an average 1.212 funded staff years in the management period.
